# The Intersection of Mitophagy and Autism Spectrum Disorder: A Systematic Review

**DOI:** 10.3390/ijms26052217

**Published:** 2025-02-28

**Authors:** Eleonora Kovacheva, Maria Gevezova, Nikolay Mehterov, Maria Kazakova, Victoria Sarafian

**Affiliations:** 1 Department of Medical Biology, Faculty of Medicine, Medical University—Plovdiv, 4000 Plovdiv, Bulgaria; eleonora.kovacheva@mu-plovdiv.bg (E.K.); mariya.gevezova@mu-plovdiv.bg (M.G.); nikolay.mehterov@mu-plovdiv.bg (N.M.); mariya.kazakova@mu-plovdiv.bg (M.K.); 2 Research Institute, Medical University—Plovdiv, 4000 Plovdiv, Bulgaria

**Keywords:** mitophagy, pathways, autism

## Abstract

Autism spectrum disorder (ASD) is a group of neurodevelopmental and biobehavioral conditions that arises from complex interactions between environmental factors and physiological development in genetically predisposed individuals. Among the most frequently observed metabolic abnormalities in ASD is mitochondrial dysfunction. Mitochondria respond to cellular stress by altering their dynamics or initiating mitophagy. In neurons, the buildup of dysfunctional mitochondria and reactive oxygen species (ROS) poses a significant risk, as these cells cannot regenerate through division. To safeguard mitochondrial health, cells rely on an efficient “clean-up mechanism” to remove compromised organelles. Mitophagy, a specific form of autophagy, is responsible for regulating the turnover of flawed and non-functional mitochondria. Impairments in this process result in the accumulation of defective mitochondria in neurons, a characteristic of several neurodegenerative disorders associated with behavioral abnormalities. This systematic review offers an in-depth summary of the present knowledge of mitophagy and underscores its pivotal role in the pathogenesis of ASD.

## 1. Introduction

Autism spectrum disorder (ASD) is one of the most significant childhood mental health conditions, characterized by its profound clinical impact, long-term outcomes, and far-reaching effects on both families and society. According to data from the Centers for Disease Control and Prevention’s Autism and Developmental Disabilities Monitoring Network, about 1 in 36 children is diagnosed with ASD (https://www.cdc.gov/ncbddd/autism/data.html accessed on 15 December 2024). The etiology at molecular, cellular, and systemic levels remains incompletely understood and is further complicated by the heterogeneity among affected individuals. Accumulating clinical evidence shows that 30% of patients with ASD have dysregulated mitochondrial function [1]. Oxidative stress is observed in energy-dependent cells such as neural, immune, and gastrointestinal [2]. Considering that mitochondria are responsible for energy supply, proliferation, apoptosis, oxidative stress, signal transduction, Ca^2+^ turnover, and iron and electrolyte homeostasis [3,4,5], their dysfunction could cause different pathophysiological conditions [6]. The buildup of misfolded proteins in the mitochondrial matrix has been shown to activate mitochondrial-specific heat shock proteins, initiating adaptive stress responses to maintain cellular homeostasis [7,8]. Although ASD is a neurobiological condition influenced by a complex interplay of genetic and environmental factors that impact brain development, its precise etiologic mechanisms remain elusive despite ongoing research.

Currently, no singular unifying cause for ASD has been identified. Neuropathological studies have uncovered significant alterations in the brains of individuals with ASD, including differences in cerebellar architecture, limbic system abnormalities, and structural changes in the cortical regions of the frontal and temporal lobes [9,10,11]. Genetic susceptibility also plays a pivotal role in ASD, as siblings of affected individuals face a higher risk compared to the general population. Furthermore, monozygotic twins exhibit a notably higher, though not absolute, concordance rate for ASD [12,13,14]. Advances in genome-wide association studies and whole-exome sequencing have expanded our knowledge for the genes associated with ASD [15]. Many of these genes encode proteins crucial for neuronal synapse function or activity-dependent neuronal processes, including regulatory proteins such as transcription factors [12,16]. Research has identified potential genetic “networks” implicated in ASD, with pathways involving neurotransmission and neuroinflammation emerging as key areas of convergence [17]. While genetic factors clearly contribute to ASD’s etiology [18], phenotypic variability remains significant, as environmental factors during prenatal, perinatal, and postnatal periods can modulate genetic risk in some individuals [19].

Impairments in mitochondrial function have been implicated as a hallmark feature in approximately 80% of individuals with ASD, underscoring its critical role in the disorder’s pathogenesis [20,21,22,23]. Given that mitochondrial dysfunction is prevalent and neuronal autophagy is impaired in the brains of ASD patients, defective mitophagy, the selective autophagic degradation of damaged mitochondria, may play a central role in ASD development [24].

Dysfunctional mitochondria can signal the induction of stress responses, including the production of mitochondrial heat shock proteins, while mitophagy works to eliminate these damaged organelles to preserve mitochondrial integrity and functionality. This intricate balance between stress response activation and mitochondrial quality control is essential for maintaining cellular and neuronal health [6]. Subsequently, defective mitophagy can result in the buildup of dysfunctional mitochondria, disturbances in calcium balance, the excessive production of reactive oxygen species (ROS), the loss of essential metabolites, and impaired ATP synthesis, all of which contribute to cellular death [25,26]. The accumulation of damaged mitochondria and ROS is especially hazardous in neurons due to their inability to regenerate through cell division. Consequently, neurons in the central nervous system (CNS) are particularly susceptible to damage [21,27,28] and depend on an efficient “cleaning mechanism” to eliminate malfunctioning mitochondria. When mitophagy is compromised, it leads to the accumulation of defective organelles in neurons, a hallmark of several neurodegenerative disorders such as Alzheimer’s disease (AD) and Parkinson’s disease (PD) [29]. In the context of ASD, the role of mitophagy remains poorly understood. The lack of therapeutic interventions underscores the need for further research into the pathophysiological mechanisms underlying this condition.

Since mitochondrial dysfunction and the accumulation of damaged mitochondria are common findings in neurodegenerative diseases, enhancing the mitochondrial clearance by increased mitophagy offers a significant therapeutic potential. Several mitophagy modulators with neuroprotective effects have been discovered so far. Some of them are used as ASD therapeutic agents like Coenzyme Q10, N-Acetylcysteine, vitamins E and C [30], vitamins from the B-group [31,32,33,34,35], the ketogenic diet and the modified Atkins diet [36], and acetyl-L-carnitine [37]. In addition, Hill et al. (2025) reported that the application of nutritional supplements, containing vitamins, minerals, and activated cofactors (SpectrumNeeds, NeuroNeeds, Old Lyme, CT, USA), in combination with the ubiquinol form of CoQ10 (QNeeds, NeuroNeeds), improved social interaction and hyperactivity, as well as lessening the efforts spent on caregiving by the parents [38].

**Figure 1:**
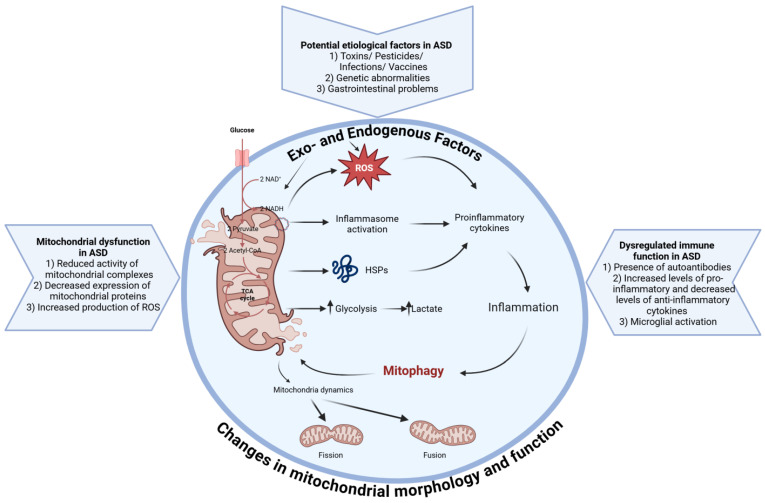
Mitochondrial quality control (MQC). Mitochondrial dysfunction, potential etiological exo- and endogenous factors, and dysregulated immune function enhance the production of reactive oxygen species (ROS) and further deteriorate mitochondria. These processes lead to inflammasome and heat shock protein (HSP) activation and an increase in glycolysis and lactate levels. Production of proinflammatory cytokines and severe inflammation follow, which enhances the clearance of damaged mitochondria via mitophagy or via fission/fusion (created with BioRender.com).

The aim of the present review is to highlight the essential role of mitophagy in the pathogenesis of ASD and its connection to neuroinflammation. Heterogeneity among affected ASD individuals necessitates the search for diagnostic biomarkers for the stratification of patients with ASD and for the selection of specific therapy. The elucidation of mitophagy dynamics can help in this, and also in establishing the relationship between clinical manifestation and disorders in mitophagy (Figure 1). The answers to these questions may reveal novel therapeutic approaches.

## 2. Methodology

### 2.1. Search Strategy

We performed a systematic review following a three-step search strategy. Initially, we conducted a comprehensive literature search across three databases, namely, PubMed, Google Scholar, and Scopus, in January 2025. Our search terms included “mitophagy”, “mitophagy pathways”, and “ASD”, allowing us to identify all studies related to mitophagy pathways and their association with ASD. This approach ensured that no relevant publications were overlooked. To focus on the most recent research, we applied a publication date filter to include articles published between 1 January 2019 and 1 January 2025. Using these keywords, our search retrieved 787 articles on mitophagy pathways and 8 articles specifically addressing the link between mitophagy and ASD across the three databases.

### 2.2. Eligibility and Study Selection

**Figure 2:**
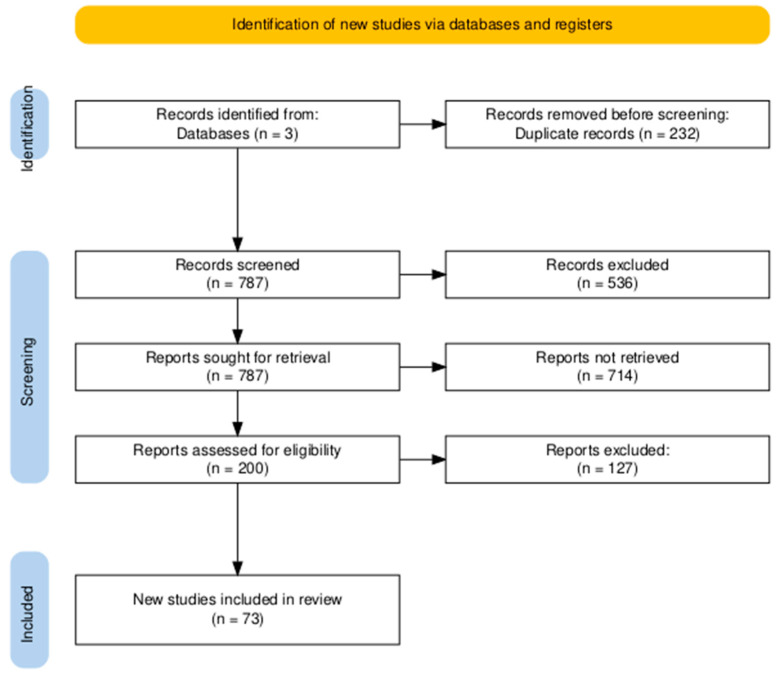
A PRISMA flow diagram illustrating the steps involved in the identification, screening, and selection of studies for inclusion [39].

The titles and abstracts of all retrieved records underwent a thorough evaluation to determine their suitability for inclusion. Studies with titles or abstracts deemed irrelevant to the research focus, along with review articles, were excluded. Subsequently, the full-text articles were meticulously examined to ensure they met the established inclusion and exclusion criteria. The following inclusion criteria were applied: (1) original research articles providing insights into the mechanisms and pathways of mitophagy; (2) original studies exploring the relationship between mitophagy and ASD; (3) studies exclusively focused on ASD; and (4) articles written in English. While this language restriction ensured uniformity, it may have resulted in the omission of relevant studies published in other languages. Exclusion criteria included (1) studies investigating mitophagy in diseases outside the scope of neurodegenerative and neurodevelopmental disorders, and (2) articles published outside the specified time frame of 2019 to 2025 (Figure 2).

## 3. The Fine Balance Between Neuronal Health and Mitophagy in ASD

Recent studies have highlighted the crucial role of mitochondrial quality control (MQC) in maintaining normal cell function and mitochondrial homeostasis [40]. It involves multilevel regulatory mechanisms that interact with each other and can be conditionally divided into two levels: molecular and organelle [41]. The response of these mechanisms differs based on the type and severity of stress to which the organelles are subjected [40]. Stressors can be either endogenous, including misfolded proteins, mitochondrial DNA mutations, and metabolic or oxidative stress, or exogenous, such as mechanical stress, infections, and hypoxia (Figure 1). Mitochondria respond to these stressors by dynamic changes or by the mitophagy-induced removal of damaged mitochondria [42,43,44]. Disruptions in MQC have been identified in various neurodegenerative disorders, including PD, AD, and Huntington’s disease (HD) [45,46,47,48], as well as in cardiomyopathies [48], eye diseases [49,50], and cancer [51,52].

**Figure 3:**
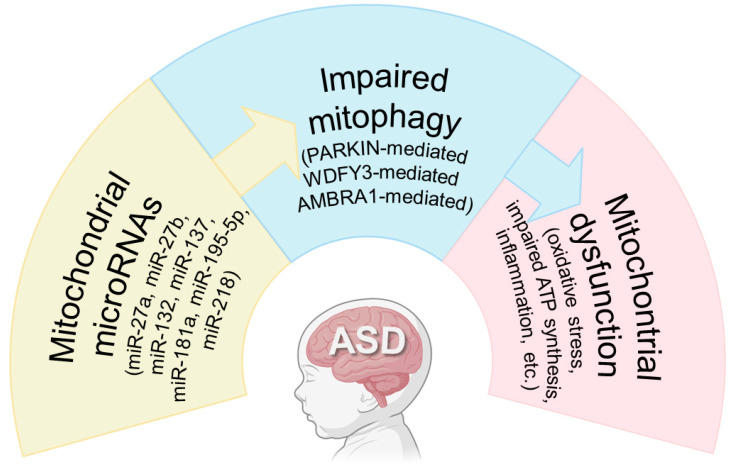
Working model integrating the mechanisms discussed within this review (created with BioRender.com).

Despite the advances at genomic, transcriptomic, and proteomic levels, it is still difficult to clearly define the pathogenesis of ASD. In recent years, increasing evidence has reported its association with mitochondrial dysfunction. It is not completely understood whether it is a mediator or moderator of disease etiology [53]. In addition, the genetic basis is still unclear, but, surprisingly, risk genes for ASD patients are associated with the mitochondrial function [54]. Interestingly, *postmortem* studies of children with ASD have revealed elevated levels of proteins linked to mitochondrial fission (such as FIS1 and DRP1), alongside a reduction in those involved in mitochondrial fusion (including MFN1, MFN2, and OPA1) [53]. The fusion of mitochondria results in the formation of single, elongated organelles, which may represent an adaptive response to increased energy demands and a sustained inflammatory oxidative environment [53]. According to Licznerski et al. (2020), mitochondria switch from immature glycolytic metabolism to mature oxidative metabolism in various cell types and brain regions in mouse neurons, leading to abnormal development [55]. Additionally, Twig (2008) observed that at the cellular level, mitochondria become fragmented and cluster around the nucleus, depriving synapses of the energy necessary to carry out their functions [56]. This accumulation of mitochondria is linked to impaired mitophagy, which prevents the removal of damaged mitochondria [56]. Structural and functional alterations in these organelles have been documented in both fibroblasts from ASD patients and in the peripheral and central nervous systems. Numerous studies have highlighted disrupted bioenergetic metabolism, with elevated levels of lactate, pyruvate, and serotonin. Changes have also been observed in respiratory complexes I, II, III, IV, and V, as well as in coenzyme Q10 levels, reduced superoxide dismutase activity, and increased oxidative damage [57,58]. Overall, mitochondrial dysfunction appears to play a significant role in the pathogenesis of ASD, primarily by disrupting the energy production essential for normal physiological processes. This dysfunction is driven either by impaired mitophagy or by excessive mitochondrial division and the accumulation of dysfunctional mitochondria (Figure 3).

## 4. Mitophagy Alterations in ASD

Over the past decade, the interest in the mechanisms regulating mitophagy has been significantly increasing. In addition to that, several mitophagy pathways have been discovered and described.

Among these, the PINK1-PARKIN pathway is the most extensively studied mechanism responsible for regulating the removal of damaged mitochondria under stressful conditions [59,60,61]. PINK1, a mitochondrial serine/threonine-protein kinase, plays a protective role by safeguarding cells against mitochondrial stress-induced dysfunction [62]. PARKIN, typically found in an inactive state within the cytosol [63,64], interacts with PINK1, which is constantly imported and degraded inside healthy mitochondria [65,66]. However, when mitochondria are damaged or depolarized, PINK1 fails to be imported into the matrix [67]. Instead, it accumulates and dimerizes on the mitochondrial surface, triggering mitophagy [68,69,70].

In addition to PARKIN, other E3 ubiquitin ligases also facilitate the removal of dysfunctional mitochondria [71]. Among these, mitochondrial ubiquitin ligase 1 (MUL1) plays a pivotal role in what is termed the PARKIN-independent ubiquitin-mediated mitophagy pathway. MUL1 interacts with four specific E2 conjugating enzymes to link damaged mitochondria to autophagosomes, initiating their degradation [72,73].

Another notable pathway is receptor-mediated mitophagy, which relies on receptors typically located on the outer mitochondrial membrane. These receptors directly interact with LC3 proteins, enabling the damaged mitochondria to be targeted by autophagosomes without requiring mitochondrial ubiquitination [74].

A fourth pathway involves lipid-mediated mitophagy. Lipids, particularly the phospholipid cardiolipin, can act as signals for the recruitment of damaged mitochondria to the autophagy machinery [75]. Cardiolipin, typically found in the inner mitochondrial membrane, migrates to the outer membrane following mitochondrial injury. This pathway operates independently of PINK1 and PARKIN. Notably, mutations in LC3 at cardiolipin-binding sites or the downregulation of cardiolipin itself significantly impair mitophagosome formation [75].

Dysfunctional mitophagy results in the buildup of damaged mitochondria, which can severely compromise neuronal function and overall brain health [76]. Increasing evidence suggests that dysfunctional mitochondria, oxidative stress, and neuroinflammation are central factors in the development of ASD [77]. Defective mitophagy is likely to play a crucial role in the disease pathogenesis, as recent studies have demonstrated that mutations in genes such as *wdfy3*, *ambra1*, and *park2* result in autism-like symptoms and mitophagy dysfunction [23,78,79] (Figure 3).

### 4.1. Park2-Mediated Mitophagy in ASD

The *park2* gene, located on chromosome 6, encodes the PARKIN protein, an E3 ubiquitin ligase responsible for tagging cellular proteins with ubiquitin to target them for degradation via the proteasome [80]. It is essential for keeping the mitochondrial health [81]. *Park2* is recognized as a critical gene associated with ASD. The ubiquitin–proteasome system is essential for maintaining synaptic function by facilitating dynamic modifications in postsynaptic density, dendritic spine structure, and synaptic terminals across both pre- and postsynaptic regions. A loss of *park2* activity can disrupt this system, leading to aberrant mitochondrial biogenesis, a process linked to ASD pathophysiology [82].

Emerging evidence highlights the significance of *park2* copy number variations in ASD pathogenesis. For instance, whole-genome studies on the *park2* gene in Chinese and European children with ASD have revealed a higher burden of copy number variations in ASD patients compared to healthy controls [83]. Moreover, studies on *park2*-deficient mice exhibit pronounced autism-like behaviors [79]. These findings strongly suggest that *park2* is a gene implicated in ASD pathogenesis, likely due to its association with mitophagy.

### 4.2. WDFY3-Mediated Mitophagy in ASD

The *Wdfy* gene family encodes four neuronally expressed intracellular proteins involved in vesicular transport. Among these, human WD repeat and FYVE domain-containing 3 (WDFY3) is a member of the BEACH (Beige and CHS proteins) family, characterized by several domains among which the FYVE domain facilitates WDFY3’s integration into vesicular membranes [84]. As an adaptor protein essential for autophagy, WDFY3 promotes the fusion of autophagic vacuoles with lysosomes. This autophagy-regulating gene plays a critical role in neurodevelopment, synaptic plasticity, and overall brain function. Research has identified *wdfy3* as a risk gene for developmental delays and intellectual disabilities [85]. Another key player, WDR40-47, is a protein associated with microtubules with a significant role in regulating autophagy and contributing to brain development. Mutations in approximately 10% of the genes encoding such proteins are linked to brain disorders, including neurodevelopmental delay and intellectual disability [86]. These findings underscore the pivotal role of WDFY3 in neurodevelopment and brain function. Furthermore, it acts as a selective autophagy adaptor that regulates mitophagy [87]. Its structural features facilitate the delivery of specific intracellular cargoes, directing impaired mitochondria to lysosomes, where they undergo degradation and removal through mitophagy. Given that maintaining mitochondrial balance is crucial for neuron differentiation, brain development, and overall brain function, mitophagy mediated by WDFY3 is likely to have an important role in the development of ASD [24].

### 4.3. AMBRA1-Mediated Mitophagy and Its Role in ASD

The activating molecule in BECLIN-1-regulated autophagy (AMBRA1) is a mitophagy receptor containing a LIR domain that facilitates direct interaction with LC3. This domain is critical in regulating both PARKIN-independent and PARKIN-dependent mitophagy pathways [88]. AMBRA1 has emerged as a key mitophagy receptor and a powerful initiator of mitophagy in mammalian cells, particularly within neurons. In the process of mitophagy, it attaches dysfunctional mitochondria to autophagosomes through its co-localization with LC3 [88]. AMBRA1 deficiency severely disrupts neuronal mitophagy [89]. Conversely, the overexpression of AMBRA1 has been shown to restore mitophagy in embryonic fibroblasts derived from PINK1 knockout mice and in fibroblasts from individuals with PD harboring mutations in PINK1 or PARKIN [90]. Beyond its role in mitophagy and apoptosis, AMBRA1 is closely linked to embryonic development, neurodevelopment, and ASD [91,92]. Research on AMBRA1 heterozygous mice reveals that they display behaviors similar to autism, including repetitive actions, cognitive inflexibility, and impaired social interactions [92]. Given AMBRA1’s crucial role in mitophagy, it is anticipated that neurons in these heterozygous mice would exhibit severely impaired mitophagy. Recent phenotype-based genetic association studies also suggest a strong link between AMBRA1 single-nucleotide polymorphisms (SNPs) and autistic traits in humans [93].

## 5. Mitochondrial microRNAs and Mitophagy

Mitochondrial microRNAs (mitomiRs) are named so due to their regulatory activity within mitochondria. These miRNAs must traverse the tightly controlled double-membrane structure of mitochondria to influence mitochondrial gene expression. Several hypotheses have been proposed regarding the mechanisms by which miRNAs gain entry into mitochondria. One of them states that AGO2 and miRNAs enter the mitochondria via sorting and assembly machinery component 50 and translocase of outer mitochondrial membrane 20 pores, followed by translocation through the inner membrane [94]. Other proteins, such as the trinucleotide repeat containing 6A and the polynucleotide phosphorylase enzyme (PNPase) are believed to facilitate miRNA import into mitochondria. PNPase, an enzyme with both 3′ to 5′ exoribonuclease and poly-A polymerase activity, resides within the mitochondrial intermembrane space and plays a critical role in regulating miRNA entry into the organelle [95]. Once inside the mitochondrial matrix, these miRNAs bind to the 3′ untranslated regions (3′UTRs) of targeted mitochondrial mRNAs, modulating gene expression. This regulation typically manifests as a downregulation of gene activity. Such effects can occur through mechanisms like mRNA degradation, reduced ribosomal binding, increased cap removal, deadenylation, and altered cap–protein interactions. The degradation of mRNA through these processes prevents it from being utilized by the translational machinery [96]. While all mitomiRs identified so far originate from nuclear DNA, there is evidence that mitochondrial DNA (mtDNA) itself can also produce miRNAs. Despite the absence of DICER and DROSHA enzymes within mitochondria, miRNAs derived from mtDNA transcripts could potentially act within mitochondria or influence other cellular compartments [94,96] (Figure 3).

A panel of ASD-related miRNAs associated with the regulation of mitophagy are presented in Table 1.

## 6. Conclusions

Considering that mitochondria are responsible for energy supply, cell growth, proliferation, apoptosis, and oxidative stress, their dysfunction could cause ATP deficiency and accumulation of ROS. The presence of damaged mitochondria and ROS in neurons is particularly dangerous because of the inability of these cells to reproduce by division. It is assumed that mitochondrial dysfunction in ASD may play a significant part in its pathogenesis, especially by disrupting the proper production of energy needed for physiological processes. This may occur through impaired mitophagy or by excessive fragmentation and the buildup of defective mitochondria.

The changes observed in patients with autism primarily affect cells with high energy demands, such as nerve, immune, and intestinal cells, providing direct evidence of a link to mitochondrial function. An obvious gap is the fact that it is not clear yet whether mitochondrial dysfunction is a cause or a consequence of ASD. Additionally, therapies involving supplements that enhance mitochondrial function are gaining popularity. They have been shown to positively impact ASD-related symptoms like social interaction and hyperactivity, and, importantly, they also reduce caregiving burden on parents. The intersection of mitophagy and ASD could serve as a basis for future research on promising potential therapeutic options.

## Figures and Tables

**Table 1:** List of mitophagy-related miRNAs in ASD.

miRNA	Function	Ref.
miR-27a and miR-27b	They modulate PINK1 expression at the translational stage, leading to a reduced efficiency of mitophagy. Specifically, miR-27a and miR-27b expression impairs processes such as ubiquitin phosphorylation, PARKIN recruitment, and the accumulation of LC3-II in damaged mitochondria.	[97]
miR-132	The dysregulation of miR-132 is linked to the pathophysiology of Fragile X Syndrome (FXS) and is a potential biomarker for the disease. In Parkinson’s patients, miR-132 causes neurotoxicity and neurodegeneration, respectively, by altering mitochondrial functions and mitophagy.	[98,99]
miR-137	This hypoxia-responsive miRNA plays a role in suppressing mitochondrial degradation through autophagy. It achieves this by directly reducing the expression of two key mitophagy receptors involved in hypoxia-driven mitophagy: FUNDC1 and NIX. Furthermore, a rare chromosomal microdeletion at 1p21.3, encompassing miR-137, has been identified in cases of ASD. Notably, the targets of miR-137 include several ASD risk genes, such as those encoding SHANK proteins (SHANK1, SHANK2, and SHANK3), which are crucial for synaptic function as well as dendritic and spine development in glutamatergic neurons.	[99,100,101]
miR-181a	It functions as a suppressor of mitophagy by targeting PARKIN expression, thereby hindering the interaction between mitochondria and autophagosomes. Conversely, studies show that silencing miR-181 is enough to enhance mitophagy in neuroblastoma cells.	[102]
miR-195-5p	It is a brain-specific ASD-associated miRNA that participates in neurodevelopment, multiple brain-related functions, and autophagy-deficiency. miR-195-5p high expression is demonstrated in rats where *bdnf* functions as a target gene.	[103]
miR-218	This miRNA targets PARKIN and has been shown to slow mitochondrial clearance in human embryonic kidney cells. It achieves this by decreasing PARKIN levels in mitochondria, reducing the ubiquitination of proteins on the outer mitochondrial membrane, and impairing the co-localization of mitochondria with LC3-II.	[104]

## Data Availability

No new data were created.
